# Osteoarthritis of the Manubriosternal Joint: An Uncommon Cause of Chest Pain

**DOI:** 10.7759/cureus.370

**Published:** 2015-11-02

**Authors:** Raju Vaishya, Vipul Vijay, Bibek K Rai

**Affiliations:** 1 Orthopaedics, Indraprastha Apollo Hospitals

**Keywords:** osteoarthritis, arthrodesis, manubriosternal joint

## Abstract

Osteoarthritis of the manubriosternal joint is a rare cause of chest pain. The diagnosis is difficult, and other serious causes of chest pain have to be ruled out first. We report one case that was treated with fusion of the manubriosternal joint using an iliac crest bone graft with a cervical locking plate and screws with excellent results. Preoperative CT scan images were used to measure the screw length and the drill stop depth. In this case report, we have shown that arthrodesis can be an effective way of treating osteoarthritis of the manubriosternal joint when other measures fail. Furthermore, the use of a cervical locking plate with appropriate and careful preoperative planning affords a safe surgical technique, rapid pain relief, and ultimately, sound and asymptomatic union of the joint.

## Introduction

Chest pain is a major cause of concern in all age groups, especially in young people, as it may indicate an underlying serious problem. Osteoarthritis (OA) of the manubriosternal joint (MSJ) is a rare cause of chest pain. The diagnosis is often difficult as other important and serious causes of pain need to be excluded before making this diagnosis. We report a rare case that was treated with fusion of the MSJ using an  iliac crest bone graft and cervical locking plate and screws with good outcome.

## Case presentation

Informed patient consent was obtained prior to surgery. No patient identifying information was included in this paper.

A 30-year-old male, farmer by occupation, presented with a history of increasing anterior chest wall pain of three years' duration. His symptoms were aggravated by exercise, sneezing, and coughing. This pain affected his ability to work and perform activities of daily living. Local examination revealed a 3 x 2 cm tender swelling over the MSJ. Terminal shoulder movements,l such as flexion and abduction, were painful and restricted. There was no associated co-morbid condition like diabetes, hypertension, or coronary artery disease.

**Figure 1:**
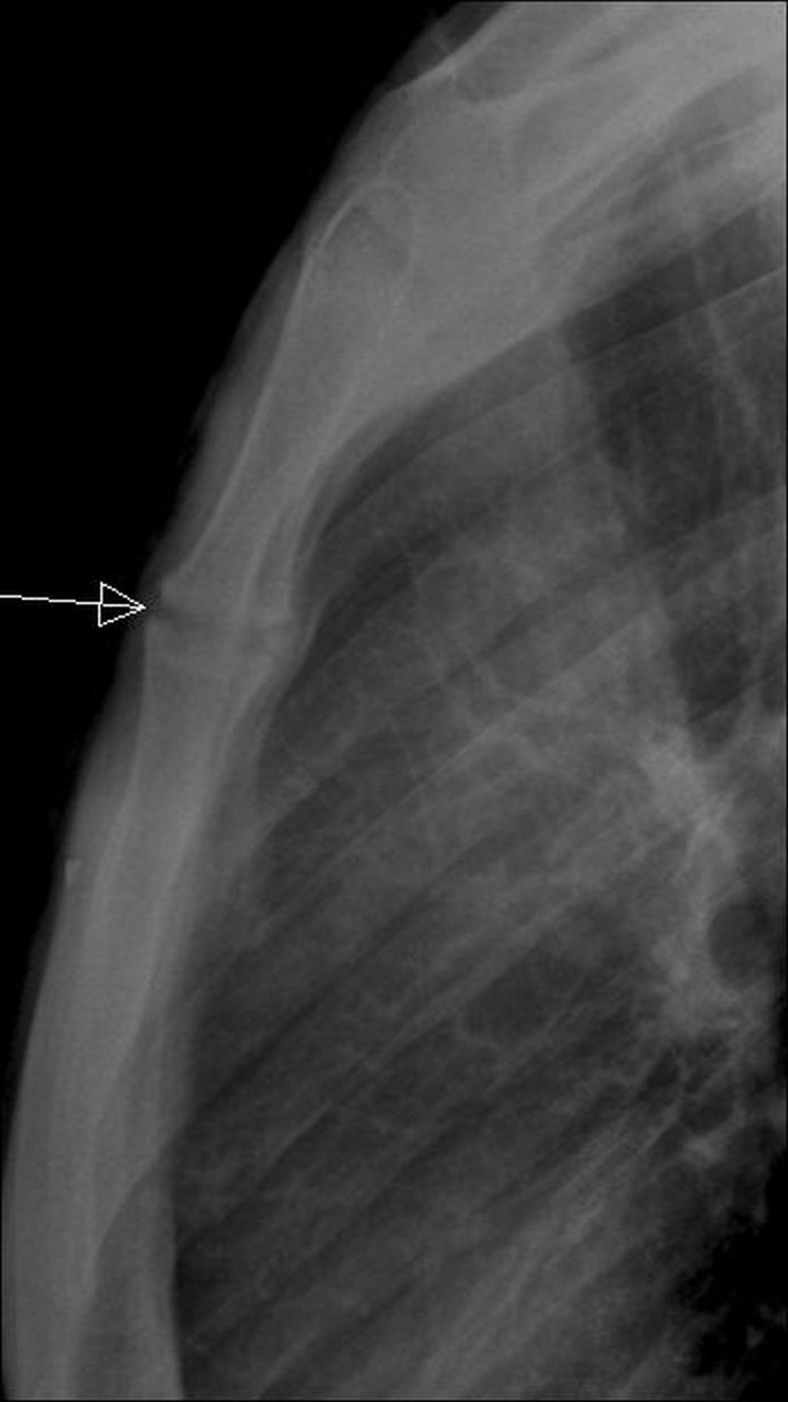
Lateral view of chest showing arthritic changes (arrow) in manubriosternal joint.

ECG and laboratory workup, including inflammatory markers like ESR and CRP, were within normal range. Lateral x-ray of the chest (sternal view) showed OA changes involving the MSJ (Figure 1).

**Figure 2:**
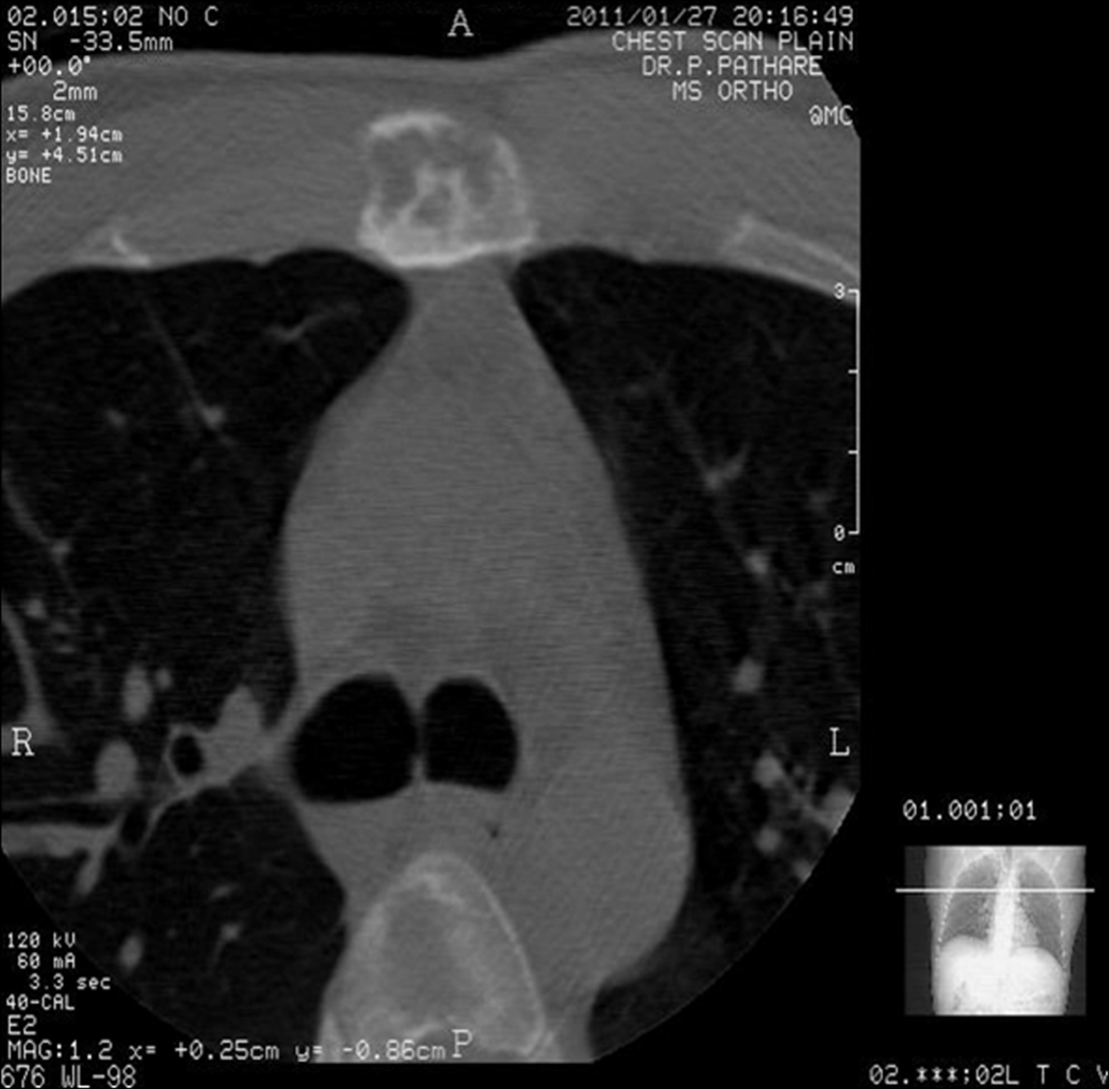
CT scan of the chest showing cavitation and multiple cyst formation in the manubriosternal joint.

The diagnosis of OA was further established by a CT scan, which also showed multiple cystic lesions (Figure 2).

The patient was treated initially by conservative means, including NSAIDs and physiotherapy, for a period of two years, but only showed symptomatic and temporary relief. An intra-articular steroid injection of 10 mg of triamcinolone was given, which resulted in some relief in pain, but the symptoms recurred. He was therefore offered the option of surgery to alleviate his persistent pain.

**Figure 3:**
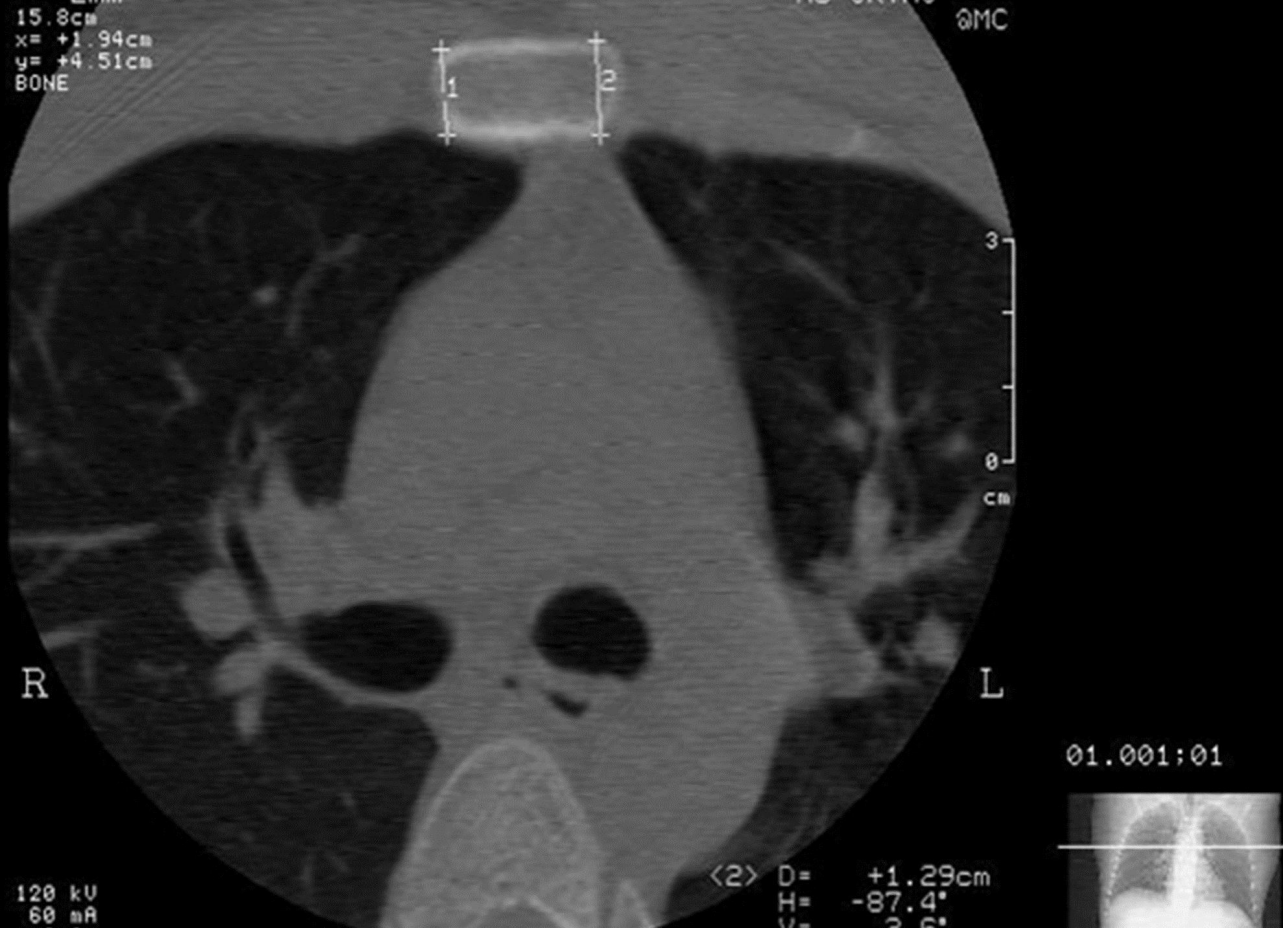
CT scan showing the measurement of thickness of manubriosternal joint.

An open reduction and internal fixation (ORIF) of the MSJ was performed under general anaesthesia in the supine position (Figure 3).

**Figure 4:**
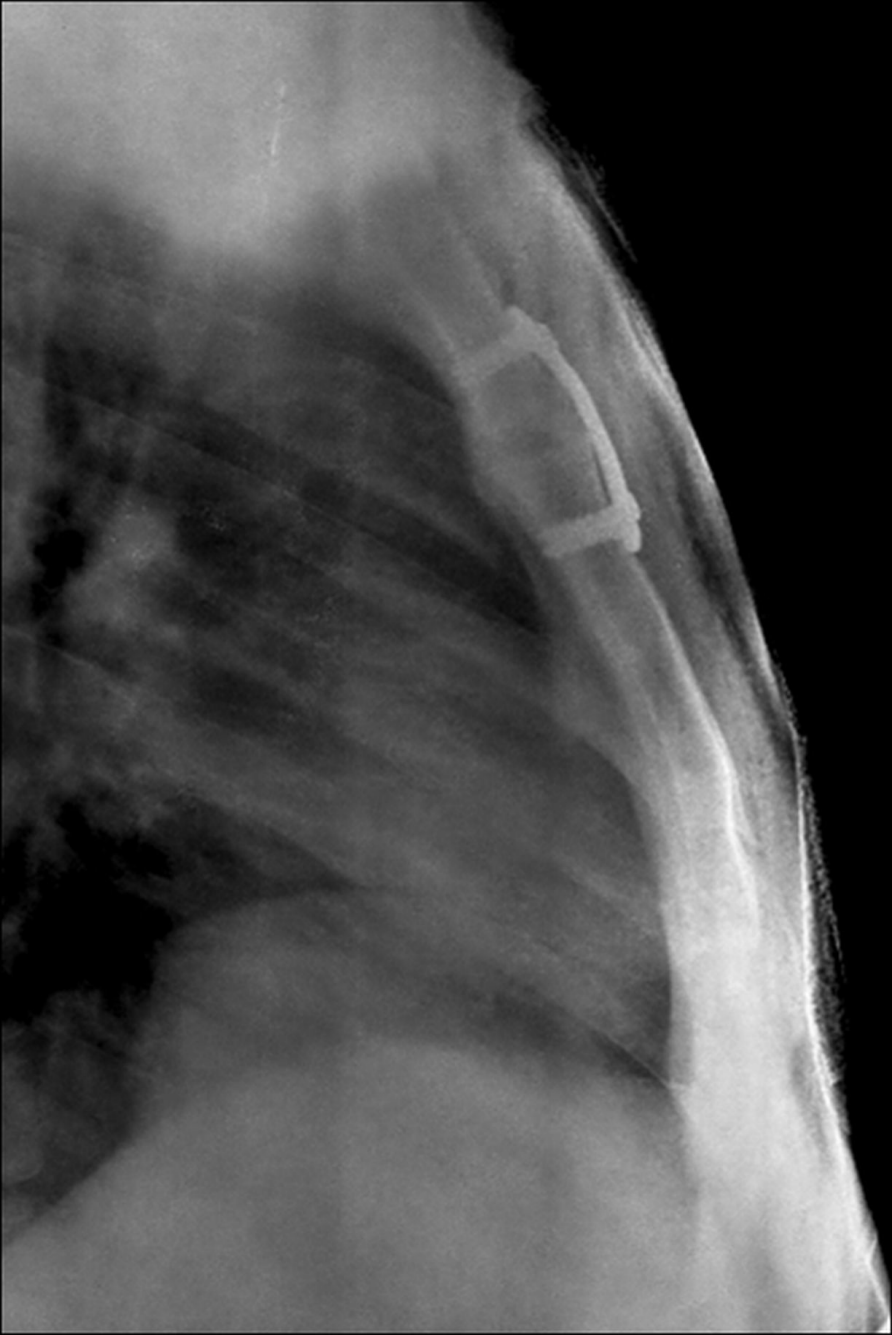
Lateral view of the chest showing fixation of manubriosternal joint with a locking cervical plate.

**Figure 5:**
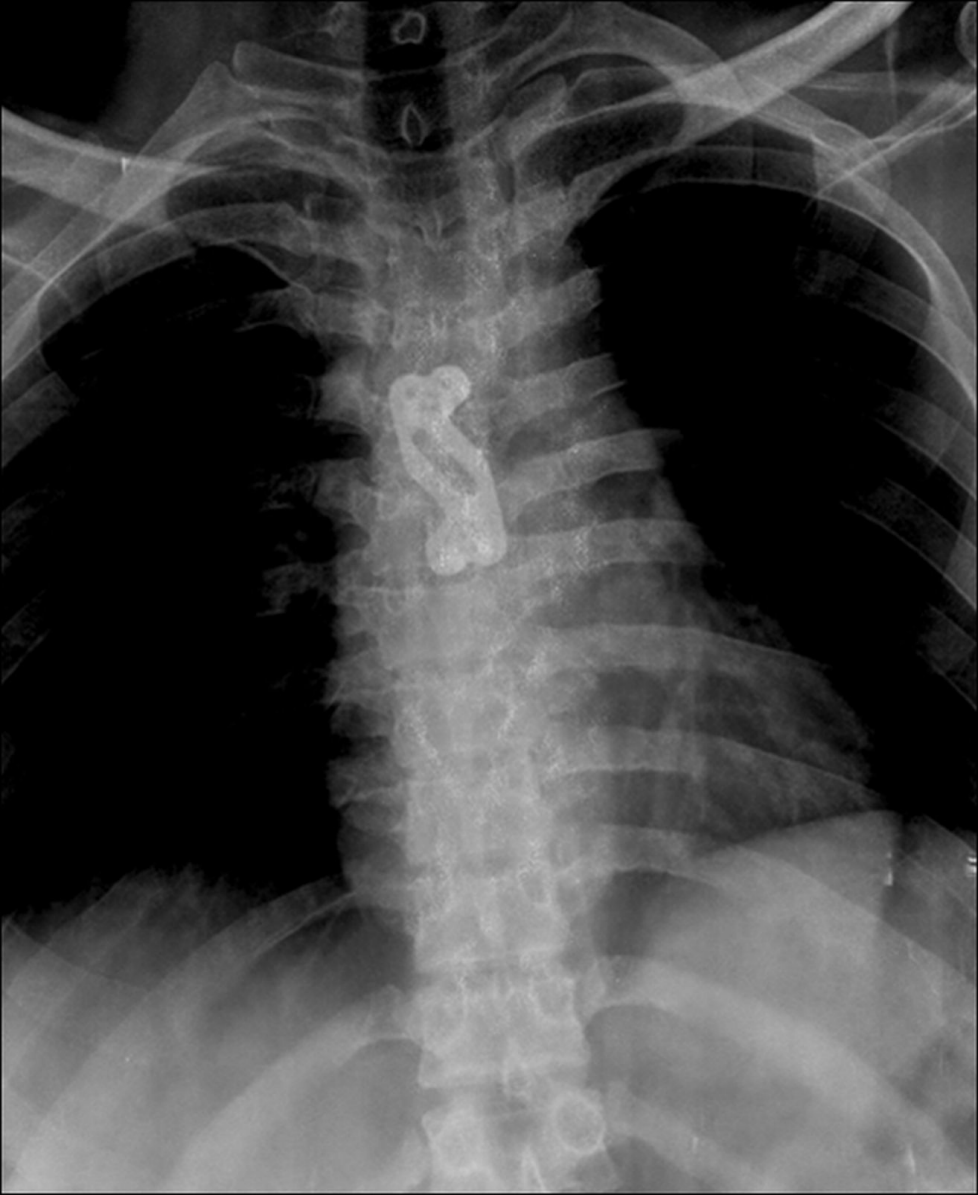
AP view of the chest showing fixation of manubriosternal joint with a locking cervical plate.

A detailed evaluation of the images of a CT scan was done prior to surgery to measure the depth of drill stop and length of screws. Surgery was done in the supine position. The exposure of the MSJ was done by an anterior midline incision. The joint was curetted out to expose the raw bleeding bony surfaces. Autologous cancellous bone graft from iliac crest was used to firmly pack the defect (Figures 4-5).

The MSJ was finally fixed with a contoured cervical locking plate, which resulted in a stable fixation of the MSJ. The curetted material sent for histopathological examination was consistent with osteoarthritis with no features suggestive of an infective etiology.

The patient was discharged on the third postoperative day. At discharge, his pain was very much relieved (Pain score: 1/10). He was advised to avoid lifting heavy weight for the initial five to six weeks following surgery. The patient resumed his activities of daily living at six weeks and returned to his heavy manual work at three months. At his two year follow-up, the patient showed sound arthrodesis of the MSJ with no pain or other discomfort.

## Discussion

Osteoarthritis of MSJ is an uncommon cause of chest pain, and hence, awareness and high index of awareness about this rare condition is crucial in reaching a diagnosis. However, other major underlying medical conditions must be ruled out before making the diagnosis and planning the operative treatment for OA of the MSJ.

An early onset of OA of the MSJ can be attributed to repetitive stress on this joint. In this patient, the repetitive stress was caused due to the lifting of heavy weights due to his profession (manual labourer) and may be the cause of the localized osteoarthritis of the MSJ. Localized symptoms and physical signs, in addition to imaging findings, often give clues to its diagnosis. Radiographic findings are often difficult to interpret, especially in early stages, and higher imaging modalities, such as CT or MRI scan, may be able to help in early diagnosis of OA of the MSJ.

The manubriosternal joint is a cartilaginous joint (symphysis) where only a thin layer of hyaline cartilage covers the articular surfaces with an intervening fibrocartilage disc. Cavitation in the disc is a common phenomenon (30%); therefore, although this joint may degenerate like a synovial joint, this joint remains a symphysis [1]. It is more commonly involved in inflammatory arthritides, including rheumatoid arthritis (RA) and gouty, psoriatic arthritis among others [2]. The symphysis at the MSJ limits anteroposterior displacement and allows only a small range of angulatory movement between longitudinal axes of the body of the sternum and manubrium. Hence, arthrits of the MSJ rarely gives rise to symptoms [2].

Arthrodesis of a joint is a known treatment option for advanced OA by various different techniques. Litchman, et al. had performed an arthrodesis in a case of post-traumatic OA of the MSJ, where they did a resection of the joint and performed a reverse sliding bone graft to fix the joint across and claimed to achieve a  ‘satisfactory outcome’. However, their patient could not return to heavy manual work postoperatively [3]. Shewring and Carvell performed an arthrodesis of MSJ for a patient with gouty arthritis, using cancellous bone grafts only (without joint fixation) [4]. Al-Dahiri and Pallister were the first to publish a case report of an arthrodesis of the MSJ for OA using double locking compression plates with good results [5]. The potential complications in the use of implants for arthrodesis of this joint include implant prominence due to the subcutaneous nature of the joint. Also, the MSJ is in close proximity to vital structures in the mediastinum and, hence, care needs to be taken to avoid injury to these structures and also ensure no possibility of implant migration. 

A locking plate provides good stability to the construct as the screws lock in the plate. Since, the MSJ cannot be immobilized firmly by a cast or a brace, the plate fixation, therefore, seems to be an excellent option of fixing the MSJ. The locking plates offer specialized advantages over normal compression plates as they have minimal chances of implant migration. Also, since the screws are locked on the plate and need not have purchase at the far cortex, smaller screws can be used for fixation. This helps in decreasing the possibility of damage to important neurovascular structures behind the far cortex, especially in places like the MSJ. These plates offer further advantages such as the very little likelihood of loosening and migration of the metal work. We preferred to use a cervical locking plate because of its thin profile to avoid hardware prominence over the sternum. Bone grafting further enhances the chance of fusion in such cases and, hence, is a preferred choice along with locking plate. Preoperative planning using the CT scan images to measure screw length and drill stop depth in order to provide safety to the underlying vital structure in this surgery was crucial.

Preoperative images of the CT scan helped us to measure the thickness of the bone and the screw length. We believe that OA of the MSJ can be treated successfully by arthrodesis when the conservative treatment fails. We believe that a cervical locking plate may be a satisfactory and safe technique for fusion, but requires good preoperative planning. The cervical locking plate provides durable pain relief and bony fusion of the MSJ.

## Conclusions

The manubriosternal joint should be considered as a differential for anterior chest pain in young males when all other differential diagnoses are ruled out. It more often affects young individuals involved in heavy manual labour. Initial management includes the use of NSAIDs and local steroid injection. In the presence of severe osteoarthritis and failed conservative means, arthrodesis should be considered. Arthrodesis using the locking plate and screws entails careful preoperative planning due to the proximity of vital structures. If well planned, the surgery can have satisfactory long-term results for this unusual diagnoses.
